# First Identification of Naturally Infected *Paratriatoma hirsuta*: A Vector for *Trypanosoma cruzi* and Implications for Chagas Disease Transmission

**DOI:** 10.4269/ajtmh.26-0007

**Published:** 2026-08-27

**Authors:** Paul A. Lenhart, Walter Roachell, Robert Fathke, Bernardo Delgado, Alexander M. Potter, Ian Copeland, Francisco Sanchez, Mauricio Solis, Amanda Cline, MaeJean Ramnarace, Gena G. Lawrence, Eyal Oren, Penelope J. Quintana, Shannon McBride, Cassandra Kerwin, Isydra G. Lujan, Tolulope Morawo, Riley E. Tedrow, Gerardo Pacheco, Jose Betancourt, Paula E. Stigler Granados

**Affiliations:** ^1^ Entomological Sciences Division, Public Health Command, San Antonio, Texas, USA;; ^2^ Entomology Branch, Walter Reed Army Institute of Research, Silver Spring, Maryland, USA;; ^3^ Food Analysis and Diagnostic Laboratory, Public Health Command, San Antonio, Texas, USA;; ^4^ Entomology Branch, Division of Parasitic Diseases and Malaria, Centers for Disease Control and Prevention, Atlanta, Georgia, USA;; ^5^ School of Public Health, San Diego State University, San Diego, California, USA;; ^6^ Navy Environmental and Preventive Medicine Unit Five, San Diego, California, USA;; ^7^ School of Health Administration, Texas State University, San Marcos, Texas, USA

## Abstract

The first detection of *Trypanosoma cruzi* (*T. cruzi*) in wild-caught *Paratriatoma hirsuta* (*P. hirsuta*) from military installations near the United States/Mexico border in California and Arizona is reported in the present study. As part of a Chagas disease surveillance initiative, 20 adult and 63 immature *P. hirsuta* were collected from 16 wood rat nests, along with sympatric *Triatoma rubida *(*T. rubida*). Initial polymerase chain reaction (PCR) screening revealed presumptive *T. cruzi*-positive samples, which were reanalyzed using a novel kinetoplast PCR assay with validated targets. Amplicons were sequenced using Sanger analysis, confirming *T. cruzi* infection. The presence of *T. cruzi* was confirmed in *P. hirsuta* (9%) and *T. rubida* (7%). The present study highlights the potential role of *P. hirsuta* as a secondary vector in sylvatic zoonotic *T. cruzi* transmission cycles in the Sonoran and Mojave Deserts and how this informs Chagas disease surveillance and prevention in the region.

Chagas disease, which is caused by the protozoan parasite *Trypanosoma cruzi *(*T. cruzi*), poses a significant public health threat across the Americas, affecting an estimated 6 to 7 million people.^1^^,^^2^ When left untreated, the disease can lead to severe chronic cardiac and gastrointestinal complications, representing a substantial burden on healthcare systems. Triatomine bugs, commonly known in the United States as kissing bugs and as chinches picudas or besucones in Mexico, are the primary vectors of *T. cruzi* to humans and other mammals. Although extensive research has been conducted on the primary vectors in endemic regions, the roles of secondary vectors in less-studied habitats remain poorly understood.^3^

More than 150 species of triatomine bugs have been described, with varying roles in the transmission of *T. cruzi*.^4^^,^^5^ Among them, the genus *Paratriatoma* includes two species. *Paratriatoma lecticularia *(*P. lecticularia*) is more widespread in the eastern United States and northeastern Mexico and has been confirmed as a vector of *T. cruzi*.^5^ In contrast, *Paratriatoma hirsuta* (*P. hirsuta*) is a rarely encountered species endemic to portions of the Mojave and Sonoran Deserts.^6^ It is most often found in woodrat (*Neotoma *spp.) nests and only occasionally in human structures.^3^^,^^6^ *Paratriatoma hirsuta* has been experimentally infected with *T. cruzi* in the laboratory but has never been detected with *T. cruzi* in the wild.^6^^,^^7^ Previous reports indicating natural *T. cruzi* infection in *P. hirsuta* are misquotes: Wood (1941) references laboratory experimental infections, Hall (1953) incorrectly attributes wild infection data from other triatomine species in the same study region, and de Paiva et al. (2021) reference *P. lecticularia* infection reports.^5^^–^^8^

Imperial County, California, and Yuma County, Arizona, are characterized by the arid western Sonoran Desert climate, sparse vegetation, and several species of wood rat.^6^ This region has not historically been considered a high-risk area for Chagas disease; however, the triatomine species found in these counties could facilitate sylvatic and peridomestic transmission cycles of* T. cruzi*.^3^^,^^9^^,^^10^

Triatomines were collected from the Chocolate Mountain Aerial Gunnery Range (CMAGR) in Imperial County, California, and the Barry M. Goldwater Range (BMGR) in Yuma County, Arizona, during field efforts in May 2021, June 2022, and September 2022 (Figure 1). These ranges are primarily used for military training by the U.S. Air Force and the U.S. Marine Corps. Entomologists from the U.S. Army Public Health Command, West (PHCW), and Navy Environmental and Preventive Medicine Unit Five collaborated with wildlife biologists from Marine Corps Air Station Yuma to conduct triatomine surveillance in woodrat nests located in sheltered areas near rocks, trees, cactus logs, or other debris. Although labor-intensive, the woodrat nest deconstruction method consistently yielded triatomines. Efforts to light-trap adults were unsuccessful. Light trapping may be successful during the brief peak in dispersing adults around July.^6^^,^^11^ Specimens were placed in individually labeled centrifuge tubes, locality data were recorded using the MAGE app (National Geospatial-Intelligence Agency, Springfield, VA; https://vector.mage.geointapps.com), and samples were frozen at −18°C. Specimens were transported on ice to PHCW and were stored at −20°C. In the laboratory, triatomines were aged and morphologically identified, and midguts were dissected from select specimens to preserve pinned morphological vouchers (see the Supplemental Information).^4^ Pinned morphological vouchers from second instar nymphs were too small for dissection and not tested for *T. cruzi *(Supplemental Information).

**Figure 1.**
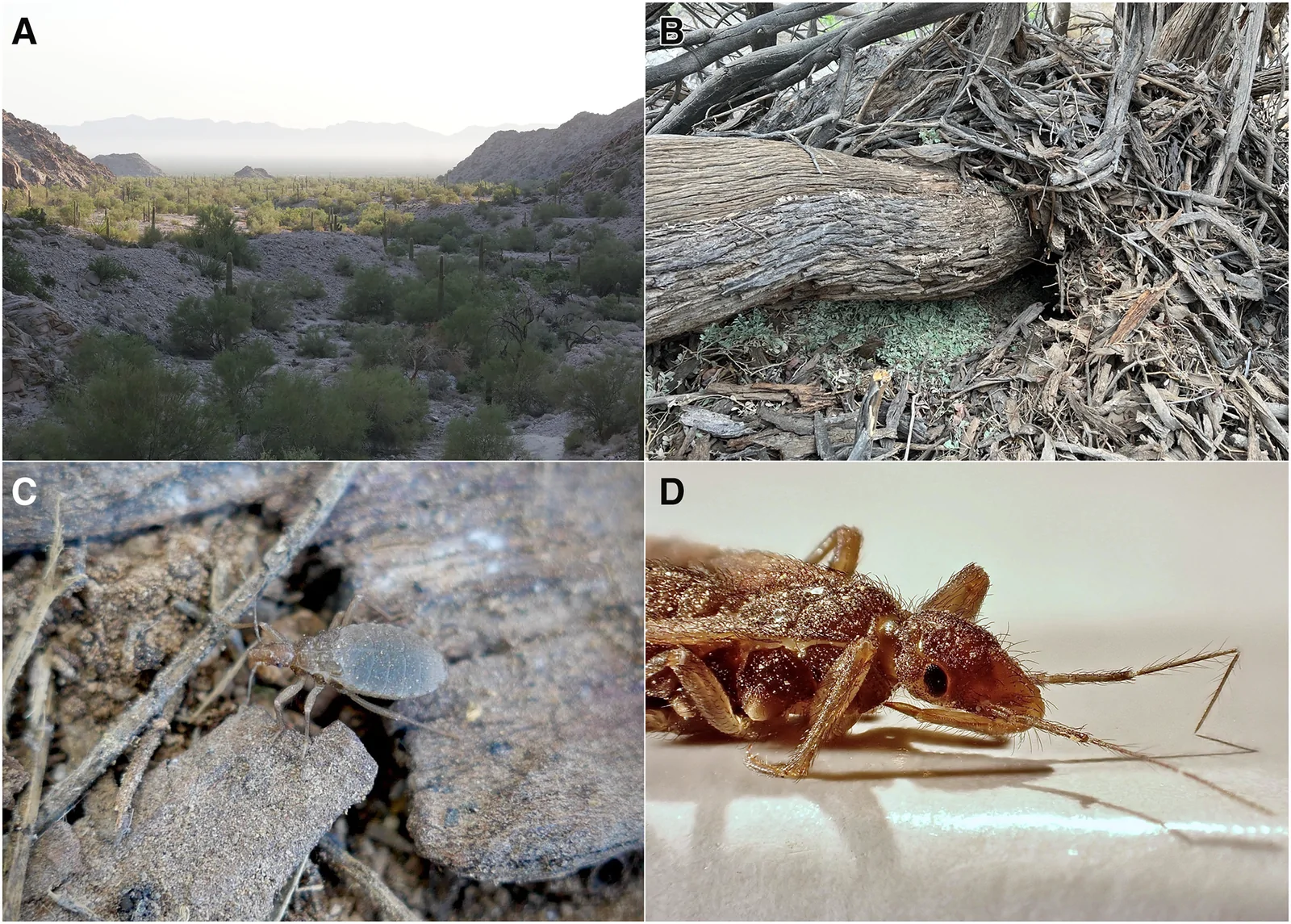
Field collection of *Paratriatoma hirsuta* (*P. hirsuta*) on the Barry M. Goldwater Range (BMGR) and the Chocolate Mountain Aerial Gunnery Range. (**A**) Typical central Sonoran Desert mountain and basin habitat in the Copper Mountains, BMGR. (**B**) Partially excavated *Neotoma* sp. woodrat nest. (**C**) Engorged fourth instar *P. hirsuta* nymph in woodrat nest debris; note the cryptic coloration. (**D**) Anterolateral view of fifth instar *P. hirsuta* nymph head and pronotum, revealing diagnostic long dark setae and an ovoid head shape.

Samples were submitted to the Department of Defense Food Analysis and Diagnostic Laboratory for molecular analysis. Results were subsequently confirmed by the Centers for Disease Control and Prevention laboratories using identical protocols. Mechanical homogenization was performed using a QIAGEN Tissue Lyser II (QIAGEN; Hilden, Germany), followed by DNA extraction using the QIAGEN DNeasy Blood and Tissue spin column (manual; QIAGEN) and the automated QIAamp 96 QIACube HT Kit (QIAGEN). Initial *T. cruzi* polymerase chain reaction (PCR) screening was conducted using the CRUZI 1–3 (Satellite DNA) and LNA 71P (kDNA minicircle) primer sets.^12^^,^^13^ Samples reactive with both primers and with a cycle threshold value <37.0 were considered “presumptive positive” and subjected to Sanger sequencing with in-house primers (Kprimer2 FWD: 5′-AGC CAT GCA TGC CTC AGA AT-3′; REV: 5′-TGC CCT CCG TAG AAG TGG TA-3′).^14^ Amplification was conducted on an ABI 7500 DX (Thermo Fisher Scientific; Waltham, MA) under the following cycling conditions: uracil-DNA glycosylase (UDG) incubation at 50°C for 2 minutes (one cycle); denaturation at 95°C for 2 minutes (one cycle); PCR at 95°C for 15 seconds, followed by 58°C for 60 seconds (40 cycles). Sanger sequencing was performed by Eurofins, targeting a 20-base sequence specific to *T. cruzi* (TGATTCAATTCATTCCGTGC).^14^

The map (Figure 2) was generated using ArcGIS Pro 3.5.0 software (Esri; Redlands, CA), and administrative boundary shapefiles were obtained from geoBoundaries (William & Mary geoLab, Williamsburg, VA; https://geoboundaries.org).^15^ The U.S. military installation boundaries were obtained from the National Transportation Atlas Database hosted by the United States Department of Transportation (Washington, DC; https://data-usdot.opendata.arcgis.com/).

**Figure 2.**
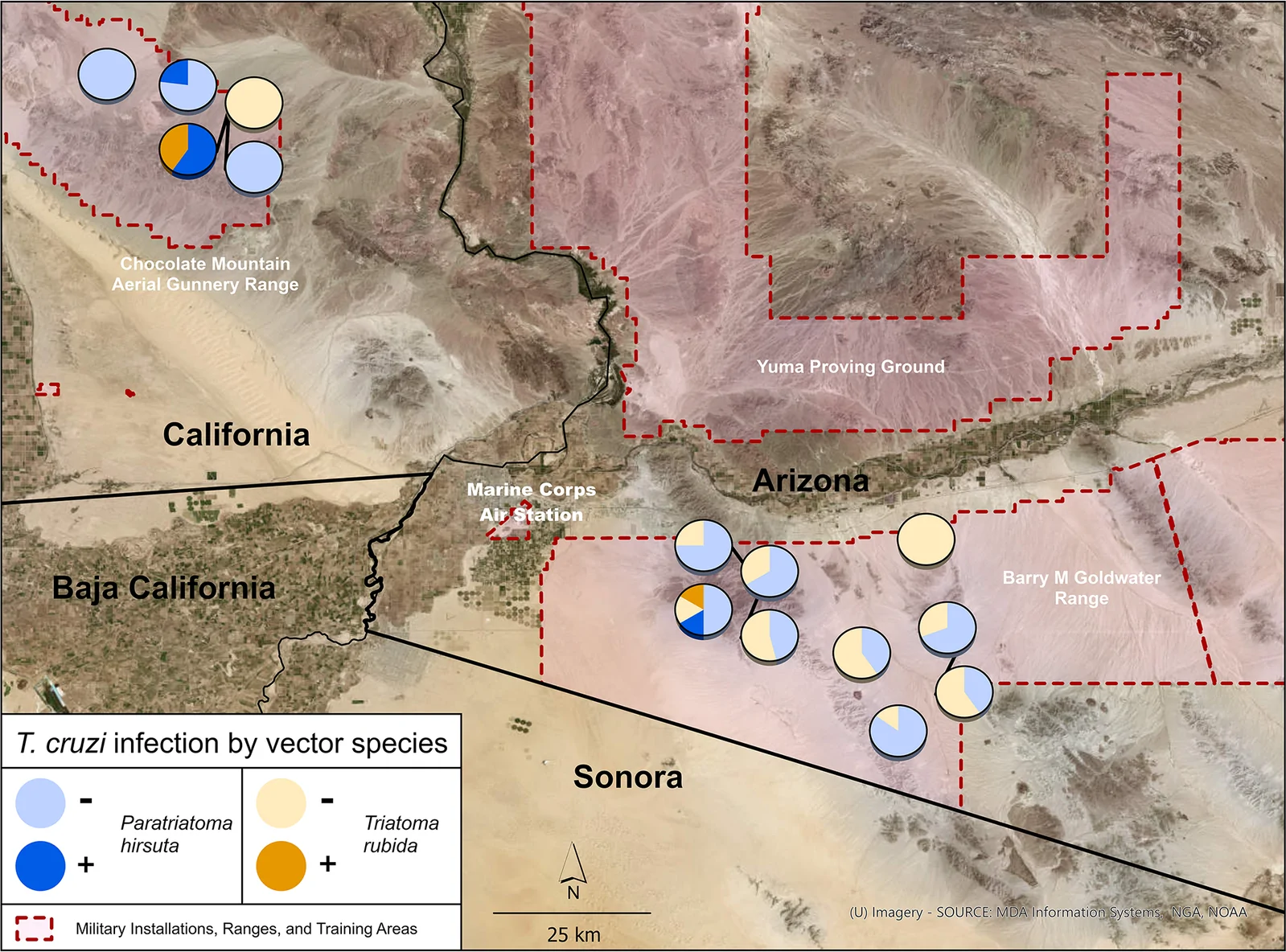
Map of collection sites on the Barry M. Goldwater Range, Arizona, and the Chocolate Mountain Aerial Gunnery Range, California, revealing proportions of *Trypanosoma cruzi*-positive and -negative kissing bugs from each sampled woodrat nest.

A total of 128 triatomines, including* P. hirsuta* and *Triatoma rubida *(*T. rubida*), were sampled from 16 woodrat nests, of which 14 were georeferenced (Figure 2 and Supplemental Information). Among the 20 adults and 63 nymphs of *P. hirsuta*, seven bugs that tested positive for *T. cruzi *were recovered from three separate woodrat nests in Arizona and California. The *T. cruzi* infection rate was 9% for *P. hirsuta* and 7% for *T. rubida.* Collections of *T. rubida* and *P. hirsuta* revealed varying levels of *T. cruzi* positivity across life stages and locality (Table 1). However, immature *P. hirsuta* and *T. rubida* at the BMGR exhibited the highest rates of *T. cruzi* at 29% and 67%, respectively. These findings should be interpreted with consideration of sample size limitations, particularly for *T. rubida *from the CMAGR, where the sample size (*n* = 3) precludes robust statistical inference about population-level infection rates.

**Table 1:** Summary of *Trypanosoma cruzi *testing results on the Barry M. Goldwater Range and the Chocolate Mountain Aerial Gunnery Range by triatomine species and life stage

Training Area	Species	Stage	Total No. Tested	No. (%)* T. cruzi*-Positive
BMGR, Arizona	*T. rubida*	Adult	9	0 (0)
		Nymph	31	1 (3)
	*P. hirsuta*	Adult	20	0 (0)
		Nymph	41	1 (2)
CMAGR, California	*T. rubida*	Adult	0	N/A
		Nymph	3	2 (67)
	*P. hirsuta*	Adult	0	N/A
		Nymph	21	6 (29)

BMGR = Barry M. Goldwater Range; CMAGR = Chocolate Mountain Aerial Gunnery Range; *P. hirsuta *= *Paratriatoma hirsuta*; *T. cruzi *= *Trypanosoma cruzi*; *T. rubida *= *Triatoma rubida.*

Infection rate percentages should be interpreted with caution, particularly for categories with small sample sizes (e.g., *T. rubida* from CMAGR: *n* = 3). Statistical comparisons are not presented because of limited statistical power.

Detecting *P. hirsuta* naturally infected with *T. cruzi* introduces critical considerations for understanding Chagas disease cycles in the Sonoran and Mojave Deserts. North America harbors 40 triatomine species across six genera, with most public health efforts historically concentrating on primary vectors. Primary vectors in Mexico include *Triatoma dimidiata*, *Triatoma gerstaeckeri *(*T. gerstaeckeri*), and phyllosoma-complex species, whereas in the United States, the primary vectors include *Triatoma sanguisuga*, *T. gerstaeckeri*, *T. rubida*, and *Hospesneotomae protracta*.^10^^,^^16^^,^^17^ However, many other species likely serve as secondary vectors. Considering its nidicolous lifestyle and distribution in the most arid North American deserts,^6^* P. hirsuta* likely plays a role as a secondary vector within sylvatic disease cycles. This is demonstrated by the presence of *T. rubida* and *P. hirsuta* found sympatrically in the same woodrat nests, with multiple individuals of both species harboring *T. cruzi *(Figure 2). Developing nymphs in woodrat nests have many opportunities for *T. cruzi* transmission over their multi-year development cycle.^4^ Direct risk to humans and domestic animals from infected *P. hirsuta* is considered lower than that from other peridomestic species for two reasons. First, although *P. hirsuta *has occasionally been reported entering human homes, these occurrences are rare compared with *T. rubida* and other synanthropic triatomines.^3^^,^^6^^,^^7^^,^^9^^,^^10^ *Paratriatoma hirsuta’s* primarily nidicolous (nest-dwelling) ecology limits opportunities for human–vector contact compared with more peridomestic species. Second, *T. rubida* exhibits more rapid post-feeding defecation than *P. hirsuta*, increasing the likelihood of parasite transmission during a blood meal event. *Triatoma rubida* defecates, on average, <2 minutes after feeding, whereas the *P. hirsuta* tested defecated 25–45 minutes post-feeding.^18^ Risk to domestic dogs is still present from ingestion of infected bugs. The demonstration of natural infection in this species, combined with occasional home invasions, indicates that exposure risk, although low, is not absent.^3^^,^^6^^,^^18^ The authors’ discovery of naturally *T. cruzi*-infected *P. hirsuta* in this portion of Arizona and California challenges existing perceptions of Chagas disease endemicity in the region.^2^^,^^3^ Infected bugs may have been missed during previous fieldwork, or it is possible that the range of *T. cruzi* is expanding in the area.

The authors of future investigations should better characterize *P. hirsuta* transmission dynamics, including blood meal analyses, the prevalence of *T. cruzi* in local mammalian reservoirs, environmental factors influencing vector distribution, and potential spillover mechanisms into domestic environments. Blood meal analysis would be particularly valuable for confirming if they are truly obligate *Neotoma* spp. feeders and assessing for any human and domestic animal feeding to refine the risk assessments in the present study. The sampling strategy, although effective for documenting *T. cruzi* infection in *P. hirsuta*, did not capture dispersing adults. Future light-trapping surveillance efforts, timed to coincide with the peak adult dispersal period in July, could provide data on the movement patterns of infected adults toward human structures. Understanding these dispersal dynamics is critical for assessing human exposure risk. The cryptic nature of this species makes it unlikely to be encountered in citizen science campaigns, which otherwise provide highly informative datasets.^19^ Large sample sizes of certain triatomines require active surveillance methods.

Military installations present unique challenges for managing vector-borne diseases in the United States.^20^ These environments often involve extensive outdoor training exercises and extended field operations, potentially increasing military personnel and working dog exposure risk to vectors in sylvatic transmission cycles. Military training frequently involves prolonged exposure to wilderness areas, overnight field exercises with bivouacking, and close proximity to sylvatic reservoir hosts, all of which may significantly elevate the risk of triatomine contact. Personnel and working dogs conducting border security operations face similar conditions and likely have a higher risk of exposure to sylvatic vectors such as *P. hirsuta*.

Given the cryptic nature of *P. hirsuta* and the unique exposure scenarios on military installations, the authors recommend implementation of targeted active surveillance strategies. These should include: 1) systematic nest surveys, deployment of shelter traps, or artificial refugia near woodrat habitat in areas proximate to military training sites and bivouac locations, particularly during preoperational site assessments; 2) light trapping efforts during peak adult dispersal periods (July) near buildings and training facilities; 3) educating military personnel and wildlife management staff on triatomine recognition and reporting; and 4) establishing protocols for testing working dogs that may have contact with sylvatic vectors. Such proactive measures would improve our understanding of vector distribution on military lands and support evidence-based risk mitigation strategies.

These findings emphasize the necessity of comprehensive, proactive surveillance strategies. Although the current risk to human and domestic animal health appears low, additional research is necessary to elucidate the ecological and epidemiological roles of secondary vectors such as *P. hirsuta*. The authors of future investigations should focus on characterizing *P. hirsuta* transmission dynamics, the prevalence of *T. cruzi* in mammalian reservoirs, environmental factors that influence vector distribution, and potential spillover mechanisms into domestic environments.

## Supplemental Materials

10.4269/ajtmh.26-0007 Supplemental Materials
