# Supplementary material for: First Identification of Naturally Infected Paratriatoma hirsuta: A Vector for Trypanosoma cruzi and Implications for Chagas Disease Transmission
Source: Am J Trop Med Hyg. 2026 Aug 27;115(4):811–5. doi: 10.4269/ajtmh.26-0007 (PMC13527189; doi:10.4269/ajtmh.26-0007)
Supplement: Supplementary file 1 [file tpmd260007.SD1.pdf]

Installation	State	County	date	morph sp	Instar	Sex	T_cruzi PCR
Barry Goldwater Marine Corps Range	Arizona	Yuma	4-May-2021	Triatoma rubida	Adult	F	Negative
Barry Goldwater Marine Corps Range	Arizona	Yuma	4-May-2021	Triatoma rubida	Adult	F	Negative
Barry Goldwater Marine Corps Range	Arizona	Yuma	4-May-2021	Paratriatoma hirsuta	Adult	F	Negative
Barry Goldwater Marine Corps Range	Arizona	Yuma	5-May-2021	Triatoma rubida	Adult	F	Negative
Barry Goldwater Marine Corps Range	Arizona	Yuma	5-May-2021	Triatoma rubida	Adult	F	Negative
Barry Goldwater Marine Corps Range	Arizona	Yuma	5-May-2021	Paratriatoma hirsuta	Adult	M	Negative
Barry Goldwater Marine Corps Range	Arizona	Yuma	5-May-2021	Paratriatoma hirsuta	Adult	F	Negative
Barry Goldwater Marine Corps Range	Arizona	Yuma	5-May-2021	Paratriatoma hirsuta	Adult	F	Negative
Barry Goldwater Marine Corps Range	Arizona	Yuma	5-May-2021	Paratriatoma hirsuta	Adult	M	Negative
Barry Goldwater Marine Corps Range	Arizona	Yuma	5-May-2021	Paratriatoma hirsuta	Adult	F	Negative
Barry Goldwater Marine Corps Range	Arizona	Yuma	5-May-2021	Paratriatoma hirsuta	Adult	M	Negative
Barry Goldwater Marine Corps Range	Arizona	Yuma	5-May-2021	Paratriatoma hirsuta	Adult	M	Negative
Barry Goldwater Marine Corps Range	Arizona	Yuma	5-May-2021	Paratriatoma hirsuta	Adult	F	Negative
Barry Goldwater Marine Corps Range	Arizona	Yuma	5-May-2021	Paratriatoma hirsuta	Adult	F	Negative
Barry Goldwater Marine Corps Range	Arizona	Yuma	5-May-2021	Paratriatoma hirsuta	Adult	F	Negative
Barry Goldwater Marine Corps Range	Arizona	Yuma	5-May-2021	Paratriatoma hirsuta	Adult	F	Negative
Barry Goldwater Marine Corps Range	Arizona	Yuma	3-May-2021	Triatoma rubida	Adult	F	Negative
Barry Goldwater Marine Corps Range	Arizona	Yuma	3-May-2021	Triatoma rubida	Adult	M	Negative
Barry Goldwater Marine Corps Range	Arizona	Yuma	3-May-2021	Paratriatoma hirsuta	Adult	F	Negative
Barry Goldwater Marine Corps Range	Arizona	Yuma	3-May-2021	Paratriatoma hirsuta	Adult	F	Negative
Barry Goldwater Marine Corps Range	Arizona	Yuma	3-May-2021	Triatoma rubida	Adult	F	Negative
Barry Goldwater Marine Corps Range	Arizona	Yuma	3-May-2021	undetermined	3		Negative
Barry Goldwater Marine Corps Range	Arizona	Yuma	6-Sep-2022	Triatoma rubida	5		Negative
Barry Goldwater Marine Corps Range	Arizona	Yuma	6-Sep-2022	Triatoma rubida	5		Negative
Barry Goldwater Marine Corps Range	Arizona	Yuma	6-Sep-2022	Triatoma rubida	4		Negative
Barry Goldwater Marine Corps Range	Arizona	Yuma	6-Sep-2022	Paratriatoma hirsuta	5		Negative
Barry Goldwater Marine Corps Range	Arizona	Yuma	6-Sep-2022	Paratriatoma hirsuta	5		Negative
Barry Goldwater Marine Corps Range	Arizona	Yuma	6-Sep-2022	Paratriatoma hirsuta	5		Negative
Barry Goldwater Marine Corps Range	Arizona	Yuma	6-Sep-2022	Paratriatoma hirsuta	5		Negative
Barry Goldwater Marine Corps Range	Arizona	Yuma	6-Sep-2022	Paratriatoma hirsuta	5		Negative
Barry Goldwater Marine Corps Range	Arizona	Yuma	6-Sep-2022	Paratriatoma hirsuta	5		Negative
Barry Goldwater Marine Corps Range	Arizona	Yuma	6-Sep-2022	Paratriatoma hirsuta	4		Negative
Barry Goldwater Marine Corps Range	Arizona	Yuma	6-Sep-2022	Paratriatoma hirsuta	4		Negative
Barry Goldwater Marine Corps Range	Arizona	Yuma	6-Sep-2022	Paratriatoma hirsuta	4		Negative

Barry Goldwater Marine Corps Range	Arizona	Yuma	6-Sep-2022	Paratriatoma hirsuta	5	Negative
Barry Goldwater Marine Corps Range	Arizona	Yuma	6-Sep-2022	Paratriatoma hirsuta	5	Negative
Barry Goldwater Marine Corps Range	Arizona	Yuma	6-Sep-2022	Paratriatoma hirsuta	5	Negative
Barry Goldwater Marine Corps Range	Arizona	Yuma	6-Sep-2022	Paratriatoma hirsuta	5	Negative
Barry Goldwater Marine Corps Range	Arizona	Yuma	6-Sep-2022	Paratriatoma hirsuta	5	Negative
Barry Goldwater Marine Corps Range	Arizona	Yuma	6-Sep-2022	Paratriatoma hirsuta	5	Negative
Barry Goldwater Marine Corps Range	Arizona	Yuma	6-Sep-2022	Paratriatoma hirsuta	5	Negative
Barry Goldwater Marine Corps Range	Arizona	Yuma	6-Sep-2022	Paratriatoma hirsuta	5	Negative
Barry Goldwater Marine Corps Range	Arizona	Yuma	6-Sep-2022	Paratriatoma hirsuta	5	Negative
Barry Goldwater Marine Corps Range	Arizona	Yuma	6-Sep-2022	Paratriatoma hirsuta	5	Negative
Barry Goldwater Marine Corps Range	Arizona	Yuma	6-Sep-2022	Paratriatoma hirsuta	4	Negative
Barry Goldwater Marine Corps Range	Arizona	Yuma	6-Sep-2022	Triatoma rubida	5	Negative
Barry Goldwater Marine Corps Range	Arizona	Yuma	6-Sep-2022	Triatoma rubida	5	Negative
Barry Goldwater Marine Corps Range	Arizona	Yuma	6-Sep-2022	Triatoma rubida	4	Negative
Barry Goldwater Marine Corps Range	Arizona	Yuma	6-Sep-2022	Triatoma rubida	4	Negative
Barry Goldwater Marine Corps Range	Arizona	Yuma	6-Sep-2022	Triatoma rubida	4	Negative
Barry Goldwater Marine Corps Range	Arizona	Yuma	6-Sep-2022	Triatoma rubida	4	Negative
Barry Goldwater Marine Corps Range	Arizona	Yuma	6-Sep-2022	Triatoma rubida	4	Negative
Barry Goldwater Marine Corps Range	Arizona	Yuma	6-Sep-2022	Triatoma rubida	4	Negative
Barry Goldwater Marine Corps Range	Arizona	Yuma	6-Sep-2022	Triatoma rubida	4	Negative
Barry Goldwater Marine Corps Range	Arizona	Yuma	6-Sep-2022	Triatoma rubida	4	Negative
Barry Goldwater Marine Corps Range	Arizona	Yuma	6-Sep-2022	Triatoma rubida	4	Negative
Barry Goldwater Marine Corps Range	Arizona	Yuma	6-Sep-2022	Triatoma rubida	3	Negative
Barry Goldwater Marine Corps Range	Arizona	Yuma	6-Sep-2022	Triatoma rubida	3	Negative
Barry Goldwater Marine Corps Range	Arizona	Yuma	6-Sep-2022	Paratriatoma hirsuta	4	Negative
Barry Goldwater Marine Corps Range	Arizona	Yuma	6-Sep-2022	Paratriatoma hirsuta	4	Negative
Barry Goldwater Marine Corps Range	Arizona	Yuma	6-Sep-2022	Triatoma rubida	4	Negative
Barry Goldwater Marine Corps Range	Arizona	Yuma	7-Sep-2022	Triatoma rubida	4	Negative
Barry Goldwater Marine Corps Range	Arizona	Yuma	7-Sep-2022	Triatoma rubida	4	Negative
Barry Goldwater Marine Corps Range	Arizona	Yuma	7-Sep-2022	Triatoma rubida	4	Negative
Barry Goldwater Marine Corps Range	Arizona	Yuma	7-Sep-2022	Triatoma rubida	4	Negative
Barry Goldwater Marine Corps Range	Arizona	Yuma	7-Sep-2022	Triatoma rubida	4	Negative
Barry Goldwater Marine Corps Range	Arizona	Yuma	7-Sep-2022	Triatoma rubida	3	Negative
Barry Goldwater Marine Corps Range	Arizona	Yuma	7-Sep-2022	Paratriatoma hirsuta	4	Negative
Barry Goldwater Marine Corps Range	Arizona	Yuma	7-Sep-2022	Paratriatoma hirsuta	4	Negative

Barry Goldwater Marine Corps Range	Arizona	Yuma	7-Sep-2022	Paratriatoma hirsuta	4		Negative
Barry Goldwater Marine Corps Range	Arizona	Yuma	7-Sep-2022	Paratriatoma hirsuta	4		Negative
Chocolate Mountain Aerial Gunnery Range	California	Imperial	8-Sep-2022	Paratriatoma hirsuta	4		Negative
Chocolate Mountain Aerial Gunnery Range	California	Imperial	8-Sep-2022	Paratriatoma hirsuta	4		Negative
Chocolate Mountain Aerial Gunnery Range	California	Imperial	8-Sep-2022	Paratriatoma hirsuta	4		Negative
Chocolate Mountain Aerial Gunnery Range	California	Imperial	8-Sep-2022	Paratriatoma hirsuta	4		Negative
Chocolate Mountain Aerial Gunnery Range	California	Imperial	8-Sep-2022	Triatoma rubida	4		Positive
Chocolate Mountain Aerial Gunnery Range	California	Imperial	8-Sep-2022	Triatoma rubida	4		Positive
Chocolate Mountain Aerial Gunnery Range	California	Imperial	8-Sep-2022	Paratriatoma hirsuta	5		Positive
Chocolate Mountain Aerial Gunnery Range	California	Imperial	8-Sep-2022	Paratriatoma hirsuta	5		Positive
Chocolate Mountain Aerial Gunnery Range	California	Imperial	8-Sep-2022	Paratriatoma hirsuta	5		Positive
Chocolate Mountain Aerial Gunnery Range	California	Imperial	8-Sep-2022	Triatoma rubida	3		Negative
Chocolate Mountain Aerial Gunnery Range	California	Imperial	8-Sep-2022	Paratriatoma hirsuta	5		Positive
Chocolate Mountain Aerial Gunnery Range	California	Imperial	8-Sep-2022	Paratriatoma hirsuta	5		Negative
Chocolate Mountain Aerial Gunnery Range	California	Imperial	8-Sep-2022	Paratriatoma hirsuta	5		Negative
Chocolate Mountain Aerial Gunnery Range	California	Imperial	8-Sep-2022	Paratriatoma hirsuta	4		Negative
Chocolate Mountain Aerial Gunnery Range	California	Imperial	8-Sep-2022	Paratriatoma hirsuta	4		Negative
Chocolate Mountain Aerial Gunnery Range	California	Imperial	8-Sep-2022	Paratriatoma hirsuta	4		Negative
Chocolate Mountain Aerial Gunnery Range	California	Imperial	8-Sep-2022	Paratriatoma hirsuta	4		Negative
Chocolate Mountain Aerial Gunnery Range	California	Imperial	8-Sep-2022	Paratriatoma hirsuta	4		Negative
Chocolate Mountain Aerial Gunnery Range	California	Imperial	8-Sep-2022	Paratriatoma hirsuta	4		Negative
Chocolate Mountain Aerial Gunnery Range	California	Imperial	8-Sep-2022	Paratriatoma hirsuta	4		Positive
Chocolate Mountain Aerial Gunnery Range	California	Imperial	8-Sep-2022	Paratriatoma hirsuta	4		Negative
Chocolate Mountain Aerial Gunnery Range	California	Imperial	8-Sep-2022	Paratriatoma hirsuta	4		Positive
Chocolate Mountain Aerial Gunnery Range	California	Imperial	8-Sep-2022	Paratriatoma hirsuta	4		Negative
Chocolate Mountain Aerial Gunnery Range	California	Imperial	8-Sep-2022	Paratriatoma hirsuta	4		Negative
Barry Goldwater Marine Corps Range	Arizona	Yuma	14-Jun-2022	Paratriatoma hirsuta	Adult	M	Negative
Barry Goldwater Marine Corps Range	Arizona	Yuma	14-Jun-2022	Paratriatoma hirsuta	Adult	M	Negative
Barry Goldwater Marine Corps Range	Arizona	Yuma	14-Jun-2022	Paratriatoma hirsuta	Adult	F	Negative
Barry Goldwater Marine Corps Range	Arizona	Yuma	14-Jun-2022	Paratriatoma hirsuta	Adult	F	Negative
Barry Goldwater Marine Corps Range	Arizona	Yuma	14-Jun-2022	Paratriatoma hirsuta	Adult	F	Negative
Barry Goldwater Marine Corps Range	Arizona	Yuma	14-Jun-2022	Paratriatoma hirsuta	Adult	F	Negative
Barry Goldwater Marine Corps Range	Arizona	Yuma	14-Jun-2022	Paratriatoma hirsuta	5		Negative
Barry Goldwater Marine Corps Range	Arizona	Yuma	14-Jun-2022	Paratriatoma hirsuta	3		Negative
Barry Goldwater Marine Corps Range	Arizona	Yuma	14-Jun-2022	Paratriatoma hirsuta	3		Negative

Barry Goldwater Marine Corps Range	Arizona	Yuma	14-Jun-2022	Paratriatoma hirsuta	3	Negative
Barry Goldwater Marine Corps Range	Arizona	Yuma	14-Jun-2022	Paratriatoma hirsuta	3	Negative
Barry Goldwater Marine Corps Range	Arizona	Yuma	14-Jun-2022	Paratriatoma hirsuta	3	Negative
Barry Goldwater Marine Corps Range	Arizona	Yuma	14-Jun-2022	Paratriatoma hirsuta	3	Negative
Barry Goldwater Marine Corps Range	Arizona	Yuma	14-Jun-2022	Paratriatoma hirsuta	3	Negative
Barry Goldwater Marine Corps Range	Arizona	Yuma	14-Jun-2022	Paratriatoma hirsuta	3	Negative
Barry Goldwater Marine Corps Range	Arizona	Yuma	14-Jun-2022	Paratriatoma hirsuta	2	Negative
Barry Goldwater Marine Corps Range	Arizona	Yuma	14-Jun-2022	Paratriatoma hirsuta	2	No data
Barry Goldwater Marine Corps Range	Arizona	Yuma	14-Jun-2022	Triatoma rubida	3	Negative
Barry Goldwater Marine Corps Range	Arizona	Yuma	14-Jun-2022	Triatoma rubida	2	Negative
Barry Goldwater Marine Corps Range	Arizona	Yuma	14-Jun-2022	Triatoma rubida	2	Negative
Barry Goldwater Marine Corps Range	Arizona	Yuma	14-Jun-2022	Triatoma rubida	2	Negative
Barry Goldwater Marine Corps Range	Arizona	Yuma	14-Jun-2022	Triatoma rubida	2	No data
Barry Goldwater Marine Corps Range	Arizona	Yuma	14-Jun-2022	Triatoma rubida	1	Negative
Barry Goldwater Marine Corps Range	Arizona	Yuma	14-Jun-2022	Triatoma rubida	Adult F	Negative
Barry Goldwater Marine Corps Range	Arizona	Yuma	14-Jun-2022	Triatoma rubida	Adult F	Negative
Barry Goldwater Marine Corps Range	Arizona	Yuma	15-Jun-2022	Triatoma rubida	3	Positive
Barry Goldwater Marine Corps Range	Arizona	Yuma	15-Jun-2022	Triatoma rubida	1	Negative
Barry Goldwater Marine Corps Range	Arizona	Yuma	15-Jun-2022	Paratriatoma hirsuta	3	Positive
Barry Goldwater Marine Corps Range	Arizona	Yuma	15-Jun-2022	Paratriatoma hirsuta	3	Negative
Barry Goldwater Marine Corps Range	Arizona	Yuma	15-Jun-2022	Paratriatoma hirsuta	3	Negative
Barry Goldwater Marine Corps Range	Arizona	Yuma	15-Jun-2022	Paratriatoma hirsuta	2	Negative
Barry Goldwater Marine Corps Range	Arizona	Yuma	14-Jun-2022	Paratriatoma hirsuta	5	Negative
Barry Goldwater Marine Corps Range	Arizona	Yuma	14-Jun-2022	Triatoma rubida	3	Negative
